# SIK2 inhibitor SIC-19 enhances the sensitivity of PARP inhibitors in triple-negative breast cancers and pancreatic cancers

**DOI:** 10.32604/or.2025.062539

**Published:** 2025-06-26

**Authors:** QIAN LI, SHUNPENG ZHU, MINGXIAN ZHU, FANG WANG, JINHUA ZHOU

**Affiliations:** 1 Department of Obstetrics and Gynecology, The First Affiliated Hospital of Soochow University, Suzhou, 215006, China; 2 Department of Pharmacology, College of Pharmaceutical Sciences, Soochow University, Suzhou, 215009, China

**Keywords:** Triple negative breast cancer (TNBC), PARP inhibitor, Salt-inducible kinase 2 (SIK2), Pancreatic cancer (PC), SIC-19

## Abstract

**Objectives:**

Our previous research demonstrated that SIC-19, an innovative inhibitor of salt-inducible kinase 2 (SIK2), effectively reduces SIK2 protein levels through the ubiquitin-proteasome pathway and exhibits synthetic lethal effects with poly ADP-ribose polymerase (PARP) inhibitors in ovarian cancer. However, the role of SIC-19 in triple-negative breast cancer (TNBC) and pancreatic cancer (PC) remains poorly defined. This study aims to investigate whether SIC-19 combined with PARP inhibitors can induce synthetic lethal effects in TNBC and PC.

**Methods:**

Cell lines with high SIK2 expression were identified through Western blot analysis. The combination’s impact was evaluated using Cell Counting Kit-8 (CCK8), clone formation, and apoptosis assays, as well as *in vivo* xenograft models.

**Results:**

Our findings indicated that the IC50 of SIC-19 was inversely correlated with endogenous SIK2 expression in TNBC and PC cell lines. SIC-19 modulates the homologous recombination repair pathway by suppressing levels of RAD50-pS635, thereby enhancing the sensitivity of TNBC and PC cells, as well as xenografts, to PARP inhibitors.

**Conclusion:**

These results underscore the potential of combining PARP inhibitors in combination with SIK2 inhibitors as a novel therapeutic approach to increase PARP inhibition’s effectiveness in treating TNBC and PC. This innovative combination therapy represents a promising approach for overcoming resistance mechanisms and improving the outcomes for patients with these challenging malignancies.

## Introduction

TNBC is a highly diverse, aggressive, recurrent, and generally poor prognostic breast cancer that accounts for about 15%–25% of all invasive breast cancer cases [1,2]. Additionally, the prevalence of pancreatic cancer is rising annually. Eighty percent of patients receive an advanced-stage diagnosis since there are no distinct symptoms in the early stage, which makes treatment very difficult [3]. Classic treatments such as surgery, radiation, and chemotherapy are often ineffective against this malignancy [4,5]. In patients with breast and pancreatic malignancies that are homologous recombination-deficient (HRD), PARP inhibitors have demonstrated promise in extending progression-free survival.

However, their efficacy is notably reduced in patients with homologous recombination-proficient tumors; the majority of these patients eventually develop resistance and experience relapse. Therefore, new intervention strategies that break through the current treatment bottleneck need to be developed to achieve a significant improvement in patient survival and clinical outcomes [2,6–9].

Poly (ADP-ribose) polymerase is one of the important components of DNA damage response (DDR). As sensors of DNA damage, they can recognize and bind to sites of DNA breaks, catalyzing the addition of ADP‒ribose units. This modification recruits other repair proteins to the damage site, thereby promoting the repair process [10–12]. In HRD cells, the inhibition of PARP leads to the accumulation of DNA double-strand breaks, ultimately resulting in the synergistic cytotoxic effects observed with PARP inhibitors (PARPis) in HRD tumors [13,14]. However, the therapeutic effectiveness of PARPis is greatly reduced in HRP-treated cells, where the DNA repair ability remains intact [7,11]. Given these challenges, enhancing the sensitivity of patients, particularly those with HRP tumors, to PARPis has become a pressing issue in the field. The development of strategies to overcome this limitation will be beneficial to expand the population of PARPis beneficiaries and improve the overall treatment outcome of patients.

As a serine/threonine kinase, salt-induced kinase 2 (SIK2) is crucial for cell signaling and can control energy metabolism. SIK2 is overexpressed in ovarian, breast, prostate, and pancreatic cancers and contributes to tumor progression [15–17]. Studies have demonstrated that SIK2 regulates cellular mitosis and that its targeted inhibition can make ovarian cancer cells more susceptible to the chemotherapy drug paclitaxel [18]. Additionally, SIK2 phosphorylates MYLK at Ser343, and p-MYLK subsequently phosphorylates MLC2, thereby modulating cytoskeletal motility and promoting ovarian cancer invasion and metastasis [19]. We previously synthesized a novel small molecule inhibitor, SIC-19, which induces SIK2 degradation via the ubiquitin-proteasome pathway. In addition to facilitating SIK2 degradation, this compound decreases RAD50 phosphorylation, impairs the nuclear translocation of RAD50, and when used with PARP inhibitors, has a synergistic cytotoxic effect on ovarian cancer [20]. While its role in breast and pancreatic cancer is not well defined, the purpose of this study was to assess the anticancer effectiveness of the SIC-19 and PARPi combination in these tumors using both *in vitro* and *in vivo* tests. Our results indicate that SIC-19 induces homologous recombination defects by downregulating DNA repair mechanisms and sensitizing HRP tumors to PARP inhibitors, thus providing new therapeutic strategies for breast and pancreatic cancers.

## Materials and Methods

### Chemicals and antibodies

Olaparib (A4154) and Niraparib (A3617) were obtained from APExBIO, Houston, United States. SIC-19 was synthesized by the American ChemBridge Compound Library. The CCK8 reagent (K1018) was also purchased from APExBIO. The CometAssay reagent kit (#4250-050-K) for single-cell gel electrophoresis assays was obtained from TREVIGEN, Gaithersburg, MD, USA. Primary antibodies used for Western blot analysis included SIK2 (#6919, 1:1000), γH2AX (#2577S, 1:1000), RAD50 (#3427, 1:1000), Phospho-Rad50 (Ser635) (#74778, 1:1000) and β-Tubulin (#2146, 1:5000). The secondary antibodies used for Western blot analysis included anti-rabbit IgG (#7074, 1:10000) and anti-mouse IgG (#7076, 1:10000). All antibodies were obtained from Cell Signaling Technology (CST), Danvers, MA, USA. Plasmids pDR-GFP (#26475) and pCBASce-I (#26477) were acquired from Addgene, Watertown, MA, USA.

### Cell culture

TNBC cell lines SUM-149-PT, HCC1806, MDA-MB-231, and MCF-7, along with the PC cell lines MIA PaCa2, BXPC3, and PANC1, were obtained from Procell Life Science & Technology, Wuhan, China. Each cell line was authenticated using STR analysis and screened for mycoplasma contamination. 10% fetal bovine serum (Pricella, 164210-50, Wuhan, China) and 1:100 penicillin/streptomycin (Basalmedia, C100C5, Shanghai, China) were added to the RPMI 1640 medium (Basalmedia, K211216) for MIA PaCa2, HCC1806, and BXPC3 or DMEM (Basalmedia, L211206) for SUM-149-PT, MCF-7, MDA-MB-231, and PANC1. The cultures were kept in an incubator with 5% CO_2_ at 37°C.

### Cell viability assay

A day beforehand, we need to plant the cells at the proper density into the 96-well plate. Five thousand cells were present in each well. Use DMSO or a series of concentrations of PARP inhibitors (Olaparib and Niraparib) and SIK2 inhibitors (SIC-19) either separately or in combination to treat the cells the following day. 10 μL of CCK8 reagent was added to each well four days later. Cell viability was assessed using a microplate reader (Biotek, SYNERGY HTX, Winooski, VT, USA) at 450 nm following additional incubation for 1–4 h at 37°C.

### Clonogenic assays

To guarantee the proper density, the cells were arranged in six-well plates with 2000 cells per well one day in advance. The medium containing the drug for the experimental group was added on the second day, and the first one was thrown away. The culture medium was taken out after the ten-day incubation period. Following two cautious PBS washes, the cells were fixed for 30 min with 4% formaldehyde (Servicebio, G1101, Wuhan, China). A suitable quantity of 0.1% crystal violet solution was added for staining for 15 min after fixing. ImageJ software (NIH, 1.8.0) was used to count colonies with 50 or more cells.

### Comet assay

Following a 72-h period of chemical treatment tailored to the experimental groups, the cells were enriched. 50 µL of the cell suspension was absorbed and dropped into the middle of the slide after 200 μL of cell suspension and molten LMAgarose (LMAI Bio, LM81351CA, Shanghai, China) were combined at a 1:10 ratio. The slides were incubated for 30 min at 4°C and then kept in the lysate for two to three hours. They were then left out of the light for an hour while incubated in the solution. After 30 min of electrophoresis at 21 volts, the slides were soaked in water for five minutes and then 70% ethanol for five minutes. After 20 min of drying at 37°C, the sample was stained with GelRed^®^ (Biotium, 41002, Fremont, CA, USA) at room temperature for 10 min in the dark. To clean the slide, immerse it in water for five minutes. It was then viewed using a fluorescence microscope (FPTRI-IX-51-2, Felle Precision Instrument, Shanghai, China) after drying fully.

### Cell apoptosis analysis

Following drug treatment by the experimental groups, the cells and cell supernatant were collected 72 h later. After centrifugation, cells were washed with PBS. After that, the cells were re-suspended in 500 μL of buffer, to which 5 μL of Annexin V-FITC and 5 μL of PI (Elabscience, E-CK-A211, Wuhan, China) were added. Flow cytometry was used to analyze the cells for apoptosis. If it cannot be detected immediately, the cell suspension should be placed on ice.

### Western blot analysis

The cells were treated according to the experimental groups. Following the predetermined time point, the cells were separated on ice for 30 min using RIPA buffer (Beyotime, P0013D, Shanghai, China), and the supernatant was then collected by centrifugation. Each sample contained 30 µg of total protein and was put onto an SDS-PAGE gel, and electrophoresis was performed at 80 V constant pressure. The proteins were then moved to a polyvinylidene fluoride (PVDF) (Epizyme, WJ002, Shanghai, China) membrane with a steady flow of 300 mA. After 90 min of closure with 5% skim milk, it was incubated for the entire night at 4°C. Then being cleaned with TBST the next day, the secondary antibody was incubated for an additional 1.5 h at room temperature. Protein expression visualization with the ECL test kit (Thermo Fisher, 32109, Waltham, MA, USA).

### Immunofluorescence staining

According to experimental groups, drugs were administered to the cells. After discarding the medium and washing it with PBS, it was fixed for 30 min at room temperature using 4% paraformaldehyde. After breaking the membranes with 1% Triton X-100 (biosharp, 21288506, Beijing, China), they were left to stand for fifteen minutes at room temperature. To lessen non-specific binding, the cells were then blocked for two hours using 5% bovine serum albumin (Macklin, A850221, Shanghai, China). This was followed by overnight incubation at 4°C with a γH2AX antibody (1:500). On the second day, anti-rabbit goat IgG (Invitrogen, 35552, Carlsbad, CA, USA) was incubated for two hours at room temperature following PBS washing. Following that, the nuclei were stained for ten minutes using 4′,6-diamino-2-phenylindole (DAPI) (CST, 4083S, Danvers, MA, USA). A Zeiss microscope (LSM980, Carl Zeiss, Oberkochen, Germany) with a 63× objective was used to take fluorescence pictures.

### Immunohistochemical staining (IHC)

The tissue samples were paraffin-embedded after being fixed for 24 h in 10% neutral buffered formalin. After cutting sections that were 4 μm thick, they were baked for two hours at 72°C, and then deparaffinized using xylene (zzstandard, ZB-092105, Shanghai, China) followed by a graded series of alcohol solutions. Sections were deparaffinized and then PBS washed three times. The sections were heated in a water bath for five minutes after being submerged in citrate buffer (10 mM, pH 6.0) and then subjected to three more PBS washes to perform antigen retrieval. The slices were incubated with 3% hydrogen peroxide for 30 min at room temperature in the dark to decrease endogenous peroxidase activity. Following additional PBS washes, the sections were incubated with 5% bovine serum albumin in 0.1% PBST for 60 min at room temperature to prevent non-specific binding sites. Sections were then incubated overnight at 4°C with primary antibodies against γH2AX (CST, #2577S, 1:200) and Ki67 (Sigma, SAB4501880, Saint Louis, MO, USA, 1:200). Following primary antibody incubation, sections were brought to room temperature, washed three times with PBS, and incubated with an HRP-conjugated rabbit secondary antibody (ImmunoWay, RS0046, Plano, TX, USA) for 60 min at room temperature. In contrast to the primary antibody, control samples underwent the same processing. Visualization was achieved using DAB+ Chromogen (Millipore, 71897, Burlington, MA, USA), and counterstaining was performed with hematoxylin (CST, 14166S). Finally, representative photos were taken after the slides were mounted and inspected under a microscope (CX33-LV2000, Olympus Corporation, Tokyo, Japan).

### HR reporter assay

Cells were inoculated one day in advance at the appropriate density in a 6-well plate, 10^5^ cells per well. On the second day, Lipofectamine 3000 (Invitrogen, L3000-015, California, USA) was used to transfect cells with the HR repair report substrate pDR-GFP plasmid and pCBASceI plasmid (no fluid change necessary). They were treated with SIC-19 for 48 h. 200 μL of frozen PBS was used to suspend the collected cells. Following mixing, flow cytometry was used to determine the percentage of GFP+ cells.

### Mouse xenograft model and treatment

We acquired female BALB/c athymic nude mice weighing 18–20 g each, aged 3–5 weeks, from GemPharmatech Co., Ltd. (Jiangsu, China). Within the animal facility, all mice were kept in an SPF setting with three-dayly bedding changes, increased water consumption, and feeding supplements. Regular personnel checks were conducted to assess the mice’s health. The nude mice received a subcutaneous injection of 1 × 10^7^ MDA-MB-231 cells into their right axilla. There were 4 treatment groups (n = 5) that the mice were randomly allocated to after five days: (a) vehicle control, (b) SIC-19 (40 mg/kg per mouse, 5 times per week), (c) Olaparib (50 mg/kg per mouse, 5 times per week), and (d) SIC-19 combined with Olaparib. Over the course of four weeks, all mice received oral treatment with either the vehicle control, a single agent, or a combination of agents. Every week, tumor sizes and body weights were recorded. The formula for calculating tumor volumes was volume = (length × width^2^)/2. The mice were put to death 35 days following treatment, and the tumors were removed for further research.

### Statistical analysis

Unless otherwise indicated, every result was conducted in triplicate, and the mean ± standard deviation (SD) is used to express quantitative data. GraphPad Prism 9 (GraphPad Software, San Diego, CA, USA) was used to plot the data, and either a 1-way/2-way ANOVA or a 2-tailed Student’s *t*-test was used for analysis. The log-rank test in GraphPad Prism was used to conduct the Kaplan-Meier survival analysis of xenograft trials. A *p*-value of less than 0.05 was considered statistically significant.

## Results

### SIC-19 inhibits endogenous SIK2 expression and increases DNA damage in TNBC and PC cells

**Figure 1:**
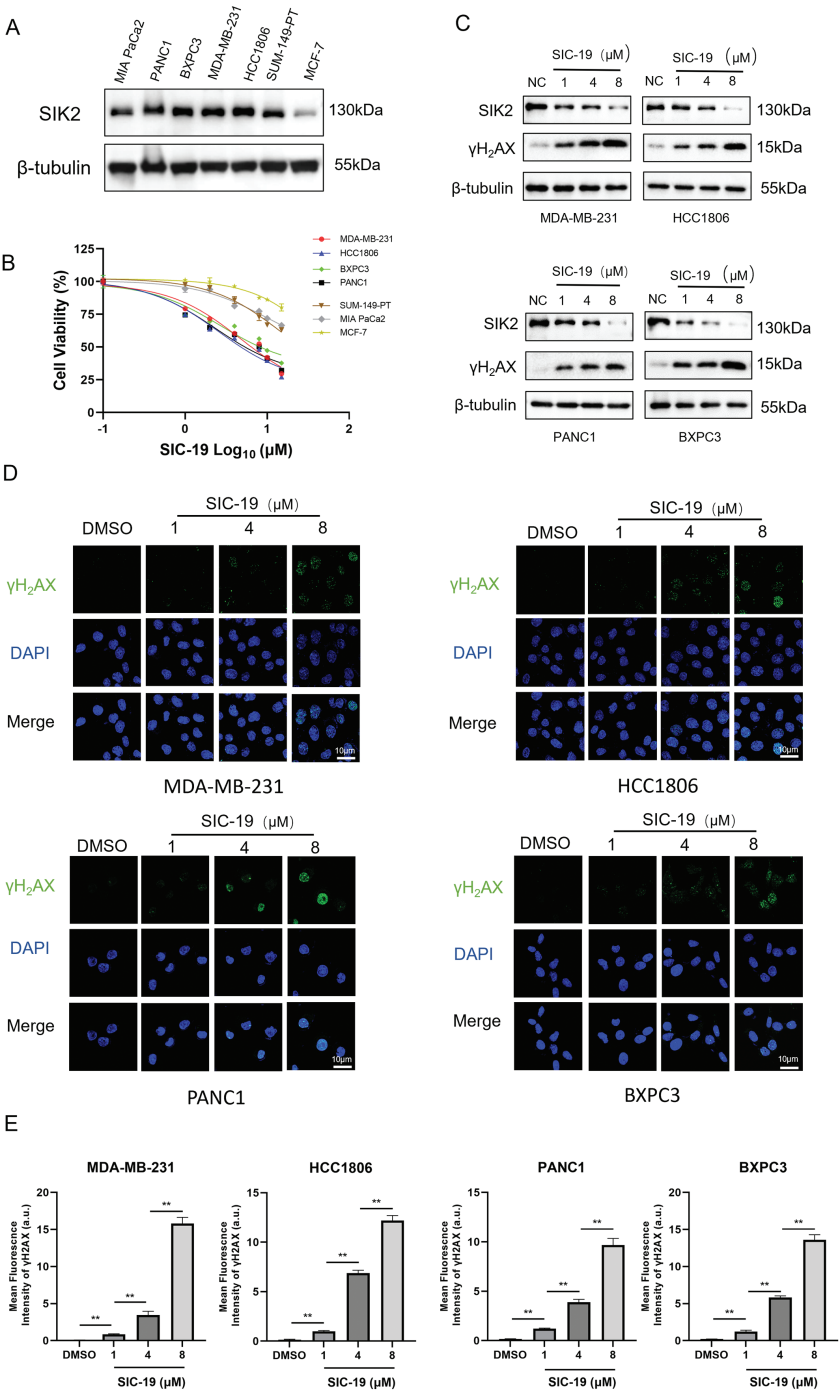
SIC-19 inhibits endogenous SIK2 expression and increases DNA damage in TNBC and PC cells. (A) SIK2 expression levels among seven cell lines. (B) Dose-response curves of SIC-19 in the seven cell lines. (C) Following 48 h of treatment with different concentrations of SIC-19, SIK2, and γH2AX cellular levels were assessed in four cancer cell lines using Western blotting. (D) Immunofluorescence (IF) analysis was used to measure γH2AX expression after 48 h of treatment with different concentrations of SIC-19. (E) Fluorescence signal statistics for each group in (D). The scale bar is 10 μm. Every data point is displayed as the mean ± SD (n = 3). **p < 0.01.

**Figure A1:**
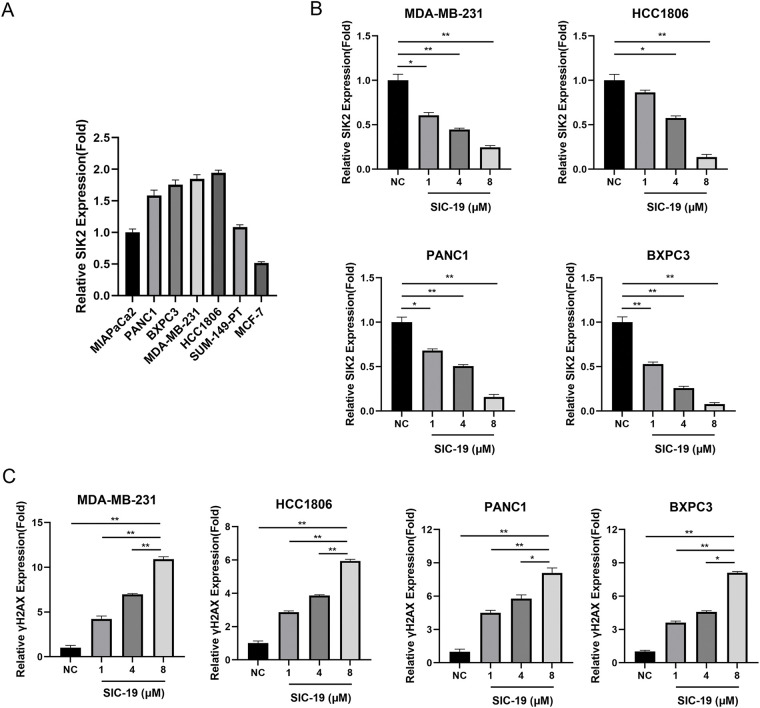
SIC-19 inhibits endogenous SIK2 expression and increases DNA damage in triple-negative breast and pancreatic cancer cells. (A) Quantification of the relative expression levels of SIK2 in the seven cell lines. (B) Quantification of the relative expression levels of SIK2 for each group in Fig. 1C. (C) Quantification of the relative expression levels of γH2AX for each group in Fig. 1C. The data are expressed as the means ± SDs of three independent experiments. *p < 0.05; **p < 0.01.

Firstly, we looked into how sensitive breast and pancreatic cancer cells were to SIC-19, a small molecule inhibitor of SIK2, when used as a monotherapy target. The IC50 values of SIC-19 ranged from 2.72 to 15.66 μM, and the cells’ sensitivity to SIC-19 increased with the level of SIK2 expression (Figs. 1A,B and A1(A)). We then selected the TNBC cell lines MDA-MB-231 and HCC1806 as well as the pancreatic cancer cell lines BXPC3 and PANC1, which have high expression of SIK2 and complete homologous recombination repair function, for experiments. Our previous studies revealed that SIC-19 targets SIK2 to induce DNA damage in ovarian cancer cells, so we next investigated the involvement of SIC-19 in triple-negative breast cancer and pancreatic cancer. Western blot analysis was used to evaluate γH2AX, a recognized indicator of DNA damage. Treatment with different concentrations of SIC-19 for 48 h strongly reduced SIK2 expression and resulted in a large increase in γH2AX levels when compared to the control group, as illustrated in Figs. 1C and A1(B,C). Moreover, γH2AX level became more pronounced with higher SIC-19 concentrations. We also evaluated γH2AX foci formation via an immunofluorescence assay. After 48 h of SIC-19 treatment, the number of γH2AX foci increased significantly (Fig. 1D,E), indicating that the cells had undergone obvious DNA double-strand breaks.

### SIC-19 inhibits HR-mediated repair and phosphorylation at RAD50-Ser635 in TNBC and PC cells

**Figure 2:**
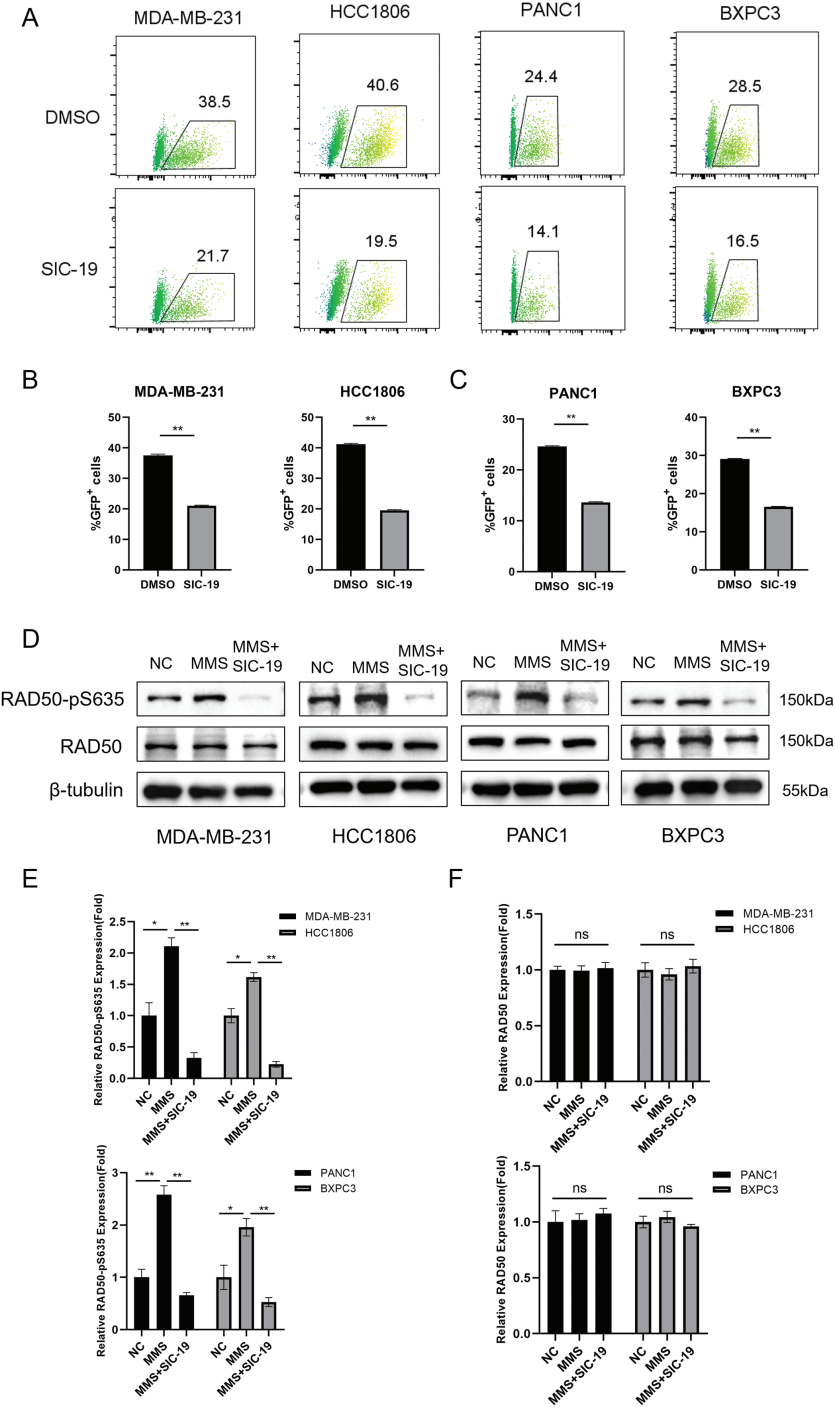
SIC-19 inhibits HR-mediated repair and phosphorylation at RAD50-Ser635 in TNBC and PC cells. (A) DR-GFP and pCBASceI plasmids were transfected into cancer cells using Lipofectamine 3000. Flow cytometry was used to measure the GFP intensity. (B and C) Statistical quantification of (A). The percentage of GFP-positive cells indicates the HR-mediated repair efficiency. (D) Total and phosphorylated RAD50 were measured by Western blot analysis in four cell lines that were exposed to SIC-19 for 48 h. (E and F) Statistical quantification of (D). Every data point is displayed as the mean ± SD (n = 3). ns: no significance; *p < 0.05; **p < 0.01.

We have demonstrated that SIC-19 can cause DNA damage in cells. Since HR is essential for repairing DNA damage, we next explored whether SIC-19 affects HR-mediated repair. Inhibiting the expression of SIK2 with SIC-19 significantly reduced the percentage of GFP+ cancer cells, and homologous recombination repair ability was weakened (Fig. 2A–C). It is well established that phosphorylation of RAD50 at Ser635 plays a significant role in DNA homologous recombination repair. Our previous research also demonstrated that SIC-19, which targets SIK2, affects DNA HR repair and increases the vulnerability of ovarian cancer cells to PARP inhibitors by inhibiting phosphorylation at RAD50-Ser635. As shown in Fig. 2D–F, following DNA damage in cells caused by methyl mesylate (MMS), the RAD50-PS635 expression level was dramatically increased. However, after the addition of SIC-19, the RAD50-PS635 expression level was considerably reduced. These results imply that via decreasing RAD50-pS635 levels, SIC-19 targeting of SIK2 impairs the capacity of TNBC and PC cells to repair homologous recombination.

### SIC-19 makes TNBC and PC cells more sensitive to PARPis in vitro

**Figure 3:**
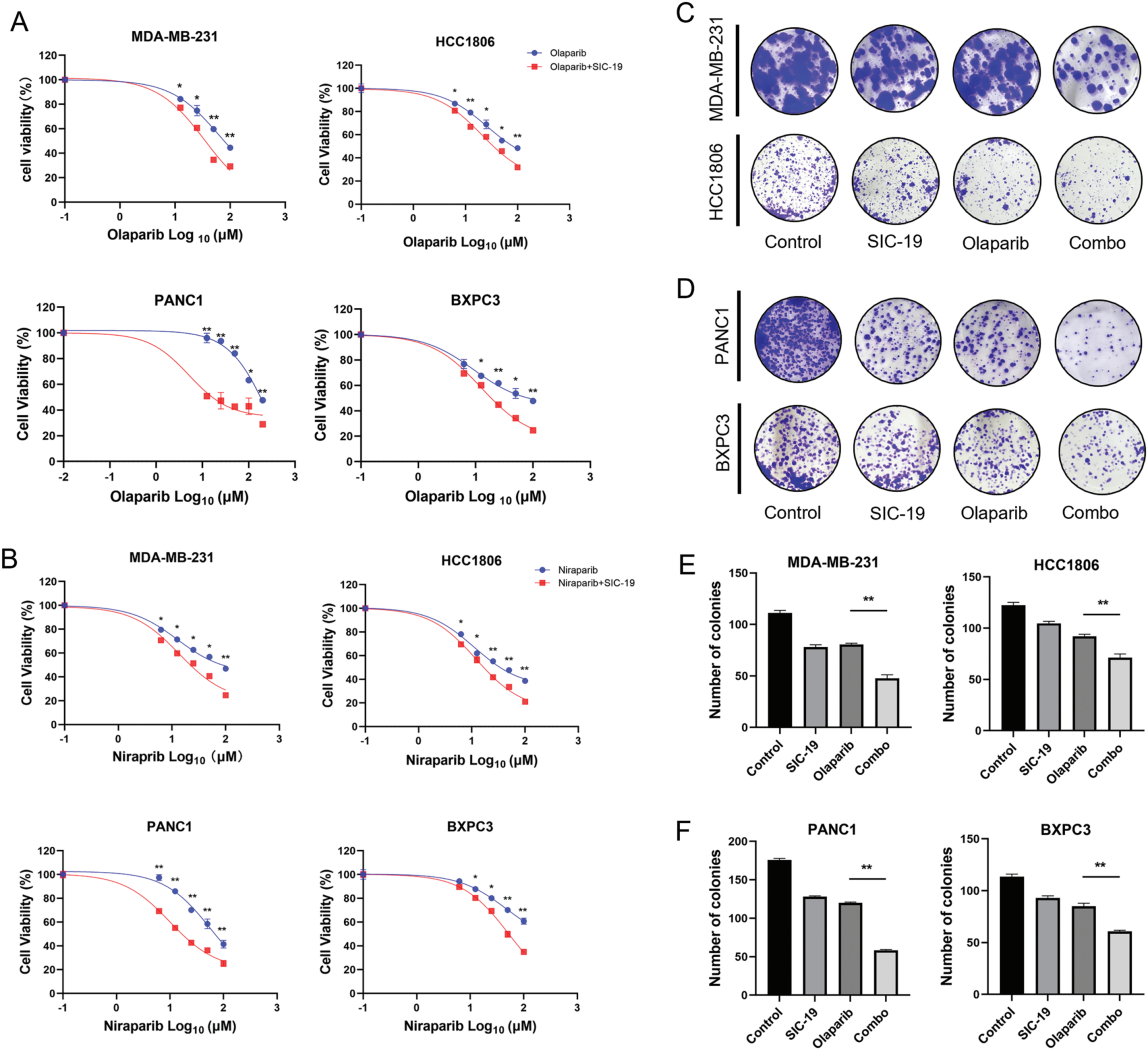
SIC-19 makes TNBC and PC cells more sensitive to PARPis *in vitro*. (A and B) Cell viability assays were performed for 96 h to assess the effects of different concentrations of olaparib or niraparib alone or in combination with SIC-19 in four cancer cell lines. (C and D) These are representative pictures of the four cancer cell lines’ clonogenicity assays. Cells were exposed to olaparib (10 μM) alone, SIC-19 (4 μM) alone, or SIC-19 plus Olaparib for 10 days. (E and F) Statistical quantification of (C–D). Every data point is displayed as the mean ± SD (n = 3). *p < 0.05; **p < 0.01.

Next, we investigated how SIC-19 affected the susceptibility of TNBC and PC to PARPis. As shown in the CCK-8 experiments, cell viability was significantly lower after the addition of SIC-19 to inhibit SIK2 than after olaparib or niraparib monotherapy, suggesting that SIC-19 makes TNBC and PC cells more susceptible to PARPis. The effects of the combinations on the tumorigenic potential of TNBC and PC cells were next assessed using colony formation assays. The combination of olaparib and SIC-19 further decreased the ability of cancer cells to proliferate when compared to monotherapy (Fig. 3C–F). These findings imply that TNBC and PC cells may become more susceptible to PARPis when exposed to SIC-19, a new small molecule inhibitor of SIK2.

### SIC-19 and olaparib cooperate to induce apoptosis and DNA damage in TNBC and PC cells

**Figure 4:**
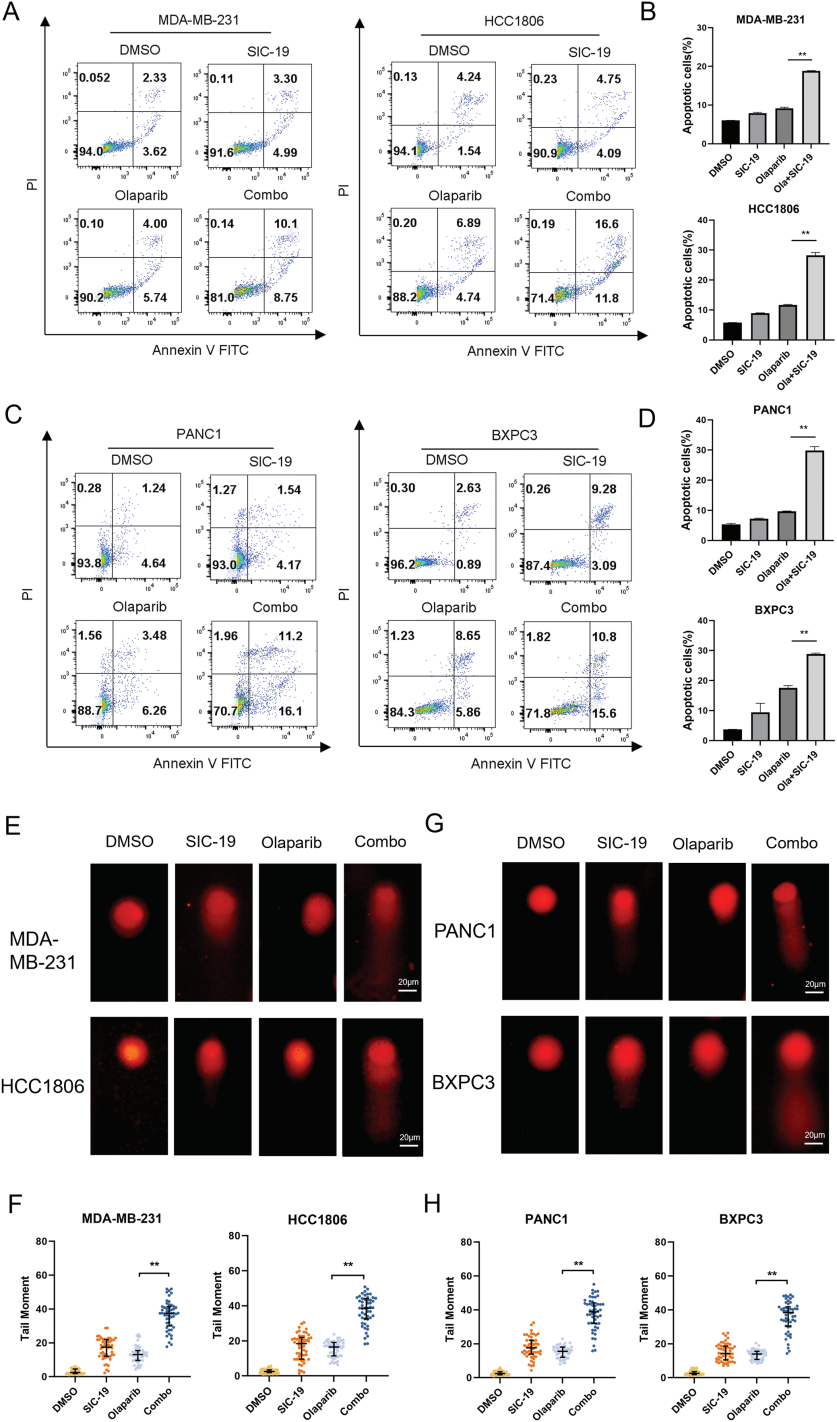
SIC-19 and olaparib cooperate to induce apoptosis and DNA damage in TNBC and PC cells. Both olaparib (10 μM for 72 h) and SIC-19 (4 μM for 72 h) were administered to all cells either separately or in combination. (A–D) The percentages of early and late apoptotic cells (B and D) as well as total apoptotic cells (A and C) are shown. (E and G) The alkaline comet assay was used to measure DNA damage in cancer cells. The scale bar is 20 μm. (F and H) Statistical quantification of (E–G). DNA damage was measured using the tail moment, which was computed using CASP software. Every data point is displayed as the mean ± SD (n = 3). **p < 0.01.

We next used Annexin V-PI labeling and flow cytometry analysis to ascertain the combination’s impact on apoptosis in TNBC and PC cells. The proportions of apoptotic TNBC and PC cells increased significantly with the combined treatment as compared to each drug alone (Fig. 4A–D). We next employed a comet assay to identify single-strand and double-strand breaks in DNA to ascertain whether the drug combination exacerbates DNA damage, as DNA damage is a major factor in apoptosis. When compared to each treatment alone, the combination of the two medications dramatically enhanced the tail intensity in cancer cells, indicating more severe DNA damage, as seen in Fig. 4E–H.

### SIC-19 combined with olaparib markedly suppressed tumor growth in MDA-MB-231 nude mouse heterotopic xenograft models

**Figure 5:**
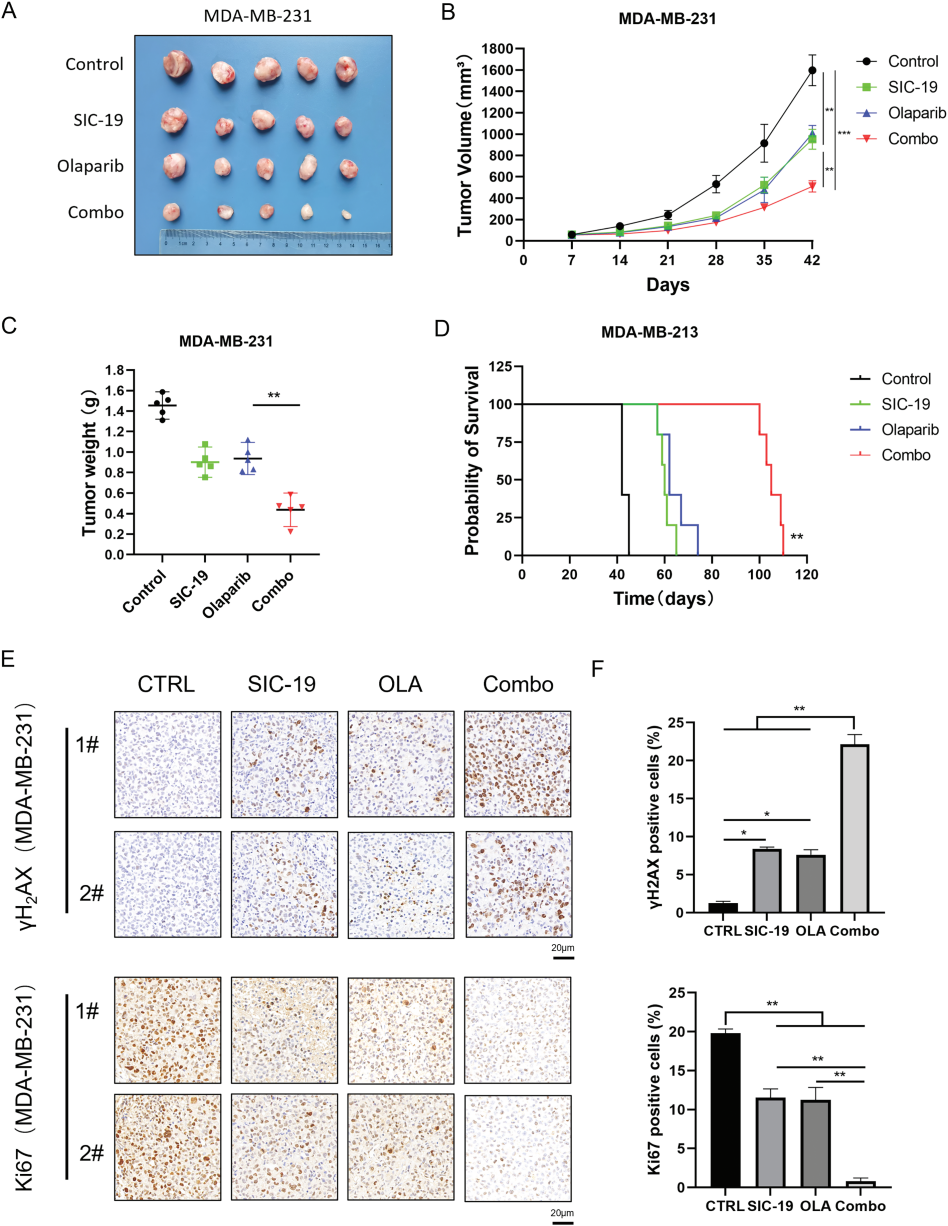
SIC-19 combined with olaparib markedly suppressed tumor growth in heterotopic xenograft models. (A–E) Nude mice with subcutaneous MDA-MB-231 tumors were treated with vehicle, SIC-19 (40 mg/kg), olaparib (50 mg/kg), or the SIC-19/olaparib combination for 35 days (n = 5 per group). (A) Photograph of xenograft tumors taken after treatment. (B) Tumor volume was calculated every seven days. (C) Tumors were weighed after the mice were sacrificed. (D) Survival of tumor-bearing mice from the start of treatment until tumors reached 1500 mm^3^ or until ethical endpoints were reached. (E) The expression of γH2AX and Ki67 in tumor tissues from MDA-MB-231 xenograft mice was assessed via immunohistochemistry; The scale bar is 20 μm. (F) Statistical quantification of (E). Every data point is displayed as the mean ± SD (n = 3). *p < 0.05; **p < 0.01; ***p < 0.001.

We further investigated the effect of SIC-19 on PARPi efficacy in an animal model. We selected the TNBC cell line MDA-MB-231, which is distinguished by strong SIK2 expression and robust homologous recombination repair function, for subcutaneous inoculation into nude mice. The model animals were treated with vehicle, olaparib, SIC-19, and the combination. Every seven days, the tumor volume was measured. After 35 days of treatment, both SIC-19 and olaparib showed some antitumor effects as single-drug treatments. The combined treatment further decreased tumor growth when compared to either drug alone (Fig. 5A–D). According to immunohistochemical examination, the group receiving SIC-19 and olaparib together had the highest amount of γH2AX expression. Conversely, the SIC-19 and olaparib cotreatment group had the lowest level of Ki67 expression (Fig. 5E,F). These outcomes support our *in vitro* findings and indicate that SIC-19 causes DNA damage in the animal model and degrades the endogenous SIK2 protein, increasing the sensitivity of breast cancer cells to olaparib.

## Discussion

PARP inhibitors have demonstrated compelling therapeutic success in breast and pancreatic cancers with defective homologous recombination repair, but they have shown only weak anticancer effects in cancers proficient in homologous recombination repair [21–23]. According to our research, TNBC and PC showed synergistic inhibition of cell proliferation when the PARP inhibitors olaparib and niraparib were combined with the SIK2 inhibitor SIC-19. Notably, cells treated with the drug combination showed the greatest levels of DNA damage and apoptosis. Additionally, olaparib and SIC-19 significantly increased the number of double-strand breaks (DSBs), one of the most harmful cell lesions. Prolonged exposure of cancer cells to genotoxic damage leads to severe structural damage to DNA, highlighting the critical role of homologous recombination in the repair process. Consequently, we investigated whether SIC-19 modifies HR to increase the effectiveness of olaparib. As shown by HR gene reporter assays, SIC-19 administration markedly reduced the cells’ ability to repair homologous recombination. RAD50 is an essential component of the MRN complex and is involved in biological processes like chromatin structure stabilization and DNA damage repair [24,25]. Moreover, phosphorylation at Ser635 of RAD50 is a recognized site involved in DNA homologous recombination repair, and we demonstrated that SIC-19, which targets SIK2, can inhibit phosphorylation at RAD50-Ser635. In addition, we evaluated the *in vivo* anticancer effects of SIC-19 in conjunction with olaparib using a subcutaneous xenograft tumor model of TNBC. Consistent with our *in vitro* findings, compared with either drug alone, the combination showed considerably higher antitumor effectiveness in the xenograft form of MDA-MB-231. Tumor tissue immunohistochemical analysis showed that SIC-19, both by itself and combined with olaparib, produced the lowest levels of Ki67, a measure of tumor cell proliferation, and the greatest levels of γH2AX, a sign of DNA damage.

Under basal conditions, SIK2 is localized in the cytoplasm. In response to DNA damage stimulation, it moves into the nucleus and settles in the vicinity of γH2AX. By directly phosphorylating RAD50 at the Ser635 site in ovarian cancer, SIK2 can influence nuclear microfilament formation and DNA recombination repair, which in turn affects sensitivity to PARP inhibitors [20]. PARP inhibitors are effective against cells that are defective in homologous recombination repair but are ineffective against cells with robust HR-mediated repair capabilities. One of the most commonly recognized mechanisms of PARPi resistance is HR-mediated repair. Thus, inhibiting HR-mediated repair is crucial to boosting PARPis’ anticancer effectiveness [26–29]. The combination of PARPis with other small-molecule inhibitors of the DNA damage response is expected to overcome resistance. Several studies have evaluated this strategy, including the use of PARP inhibitors in conjunction with CDK1 inhibitors (CDK1is), which inhibit BRCA1 phosphorylation, as well as the combination of PARPis with BET inhibitors (BETis), which downregulate BRCA1 and RAD51 expression [30,31]. These combinations increase the cytotoxicity of PARPis by disrupting homologous recombination repair. In addition, by inhibiting HR, the PI3K inhibitor BKM120 increased the glioblastoma’s sensitivity to the PARP inhibitor rucaparib [32]. In a CARM1-dependent way, EZH2 inhibitors can increase MAD2L2 and make HR-proficient ovarian cancer more sensitive to PARPi [33]. According to our findings, SIK2 inhibitors may make HR-proficient tumors more sensitive to PARP inhibitors. Additionally, as HR deficiency might make cancer cells more susceptible to treatments that damage DNA, SIK2 inhibitors can work in concert with conventional chemotherapeutic drugs. Our research offers a solid theoretical foundation for the combination of SIK2 inhibitors with other DNA-damaging anticancer agents. Previous research has demonstrated that SIC-19 can degrade SIK2 through the ubiquitin-proteasome pathway, and the specific degradation mechanism will be further explored in the future.

## Conclusion

In conclusion, we demonstrated that the growth of TNBC was successfully suppressed both *in vitro* and *in vivo* by the combination of PARP inhibitors and the SIK2 inhibitor SIC-19. This combination synergistically induced apoptosis and DNA damage in TNBC and PC. SIC-19 decreases the ability of cancer cells to repair homologous recombination by blocking the phosphorylation of the RAD50-Ser635 site, which makes cancer cells more susceptible to PARPis. Our results indicate that combining SIC-19 with PARP inhibitors may be a promising therapeutic approach for treating TNBC and PC. Nevertheless, additional research is required to validate these results.

## Data Availability

Data supporting the study’s conclusions can be found in the article.
